# Supplementary material for: Dynamics of stunting from childhood to youthhood in Ethiopia: Evidence from the Young Lives panel data
Source: PLoS One. 2020 Feb 7;15(2):e0229011. doi: 10.1371/journal.pone.0229011 (PMC7006942; doi:10.1371/journal.pone.0229011)
Supplement: Attachment — Submitted filename: Reviewer comments.docx [file pone.0229011.s001.docx]

**Review comments**

Dear Journal Editor,

Thank you for inviting me to review this manuscript: “**Dynamics of stunting from childhood to youthhood in Ethiopia: Evidence from the Young Lives panel data (rounds 1-5)”**

The paper addresses a pressing public health challenge in the study setting and of importance.

**General comments**

1. The author defined stunting and its global and national burden, trends, and determinants. However, upstream and downstream factors contributing to the steady decline in stunting over years were nor addressed in detail. It would give better understanding about the epidemiology of stunting for the readers of the manuscript if the author could include structural and other factors that have contributed to a disproportionate burden of stunting between regions within the country.

2. The author reported one of the interventions to ensure food security and combating stunting such as Productive Safety Net program; however, the time periods for the intervention was not mentioned. Were the study area, kebeles or study subjects beneficiaries of the intervention? Is there information about how long the households were included in the program? If so, the data acquisition process should be reported. In comparison of stunting among households with PSNP, did the author looked for the presence of other similar interventions in the area that could increase the effect size in reducing stunting.

**Materials and methods**

3. Who did collect the data? The author should have clearly described how the data were obtained. Who did a follow-up of the cohorts? Though the data sources were indicated in the given references, the author did not describe the cohort profile for the study subjects selected for the study (number/proportion of loss-to-follow up due to various reasons, deaths etc).

**Discussion**. The limitations of the study should be discussed in more detail.
